# Inhibition of PI3K Class IA Kinases Using GDC-0941 Overcomes Cytoprotection of Multiple Myeloma Cells in the Osteoclastic Bone Marrow Microenvironment Enhancing the Efficacy of Current Clinical Therapeutics

**DOI:** 10.3390/cancers15020462

**Published:** 2023-01-11

**Authors:** Hugh Kikuchi, Eunice Amofa, Maeve Mcenery, Steve Arthur Schey, Karthik Ramasamy, Farzin Farzaneh, Yolanda Calle

**Affiliations:** 1 Department of Haemato-Oncology, King’s College London, London SE5 9NU, UK; 2 Department of Haematology, Guys Hospital, Guys and St. Thomas’ NHS Foundation Trust, London SE5 9RS, UK; 3 Royal Berkshire Hospital, Oxford University Hospitals, Oxford OX3 7LE, UK; 4 School of Life Sciences and Health, University of Roehampton, London SW15 4JD, UK

**Keywords:** myeloma, tumour microenvironment, co-culture system, dexamethasone, resistance, osteoclasts, high throughput screening

## Abstract

**Simple Summary:**

The activation of osteoclasts occurs in up to 80% of myeloma patients. Osteoclasts promote myeloma resistance to therapeutic treatments and the formation of bone lytic lesions, resulting in bone pain and fractures in patients. However, the role of osteoclasts in drug resistance is generally overseen when performing preclinical screenings to identify new therapeutics for multiple myeloma. We propose in our study a new methodology, scalable to high throughput for the analysis of drug efficacy to better predict efficacy in myeloma patients that develop bone disease. We have identified GDC-0941 as a drug candidate that, used in combination treatments, could enhance the efficacy of currently used therapeutic drugs for myeloma by overcoming resistance mediated by osteoclasts, as well as bone marrow mesenchymal/fibroblastic stromal cells. Our work supports exploring GDC-0941 in combination with current clinical drugs for myeloma patients with active bone disease.

**Abstract:**

Osteoclasts contribute to bone marrow (BM)-mediated drug resistance in multiple myeloma (MM) by providing cytoprotective cues. Additionally, 80% of patients develop osteolytic lesions, which is a major cause of morbidity in MM. Although targeting osteoclast function is critical to improve MM therapies, pre-clinical studies rarely consider overcoming osteoclast-mediated cytoprotection within the selection criteria of drug candidates. We have performed a drug screening and identified PI3K as a key regulator of a signalling node associated with resistance to dexamethasone lenalidomide, pomalidomide, and bortezomib mediated by osteoclasts and BM fibroblastic stromal cells, which was blocked by the pan-PI3K Class IA inhibitor GDC-0941. Additionally, GDC-0941 repressed the maturation of osteoclasts derived from MM patients and disrupted the organisation of the F-actin cytoskeleton in sealing zones required for bone degradation, correlating with decreased bone resorption by osteoclasts. In vivo, GDC-0941 improved the efficacy of dexamethasone against MM in the syngeneic GFP-5T33/C57-Rawji mouse model. Taken together, our results indicate that GDC-0941 in combination with currently used therapeutic agents could effectively kill MM cells in the presence of the cytoprotective BM microenvironment while inhibiting bone resorption by osteoclasts. These data support investigating GDC-0941 in combination with currently used therapeutic drugs for MM patients with active bone disease.

## 1. Introduction

Multiple myeloma (MM) remains an incurable haematological malignancy despite major advances in the development of new therapeutic agents and the improvement in survival. The battery of drugs and therapeutic combinations available against MM has largely expanded in the last two decades with the introduction of the next generation proteasome inhibitors carfilzomifb and ixazomib, the immunomodulators lenalidomide and pomalidomide, the histone deacetylase inhibitor panobinostat, as well as newly developed antibody based therapies elotuzumab and daratumumab [1,2,3], bispecific antibodies targeting BCMA, GPRC5d, and FcRH5, and cell immunotherapy [4]. However, at present no approved personalised treatment pathways exist based on patterns of genetic abnormalities, the disease stage, and the efficacy of a particular type or sequence of drug treatments [2,3]. Additionally, the great majority of MM patients will relapse and undergo progression and currently there are no specific biomarkers that can predict the best therapy to intervene at this disease stage [1,5]. Hence, there is an urgent unmet need to improve treatments against MM, and developing new more efficacious therapies will require a deeper understanding of MM biology and the mechanisms of drug resistance.

The development of specific supportive conditions in the BM microenvironment through the interaction of MM cells with the BM stroma are critical for MM-cell growth, survival, enhanced MM invasive capacity, and resistance to therapy [6,7,8]. The effects of the newer drugs in MM have been related, in part, to their direct impact not only on MM viability, but also on the protective BM cells [1,6,8]. However, MM tumours eventually become resistant to these treatments and the exact signalling pathways or the specific cellular or molecular cytoprotective BM niches involved in this process remain elusive [2,5,9,10]. The signalling cascades associated with drug resistance may be triggered not only in MM cells, but also in BM stromal cells as a result of the direct impact of treatments. When administered in patients, cytotoxic drugs with high cancer cell killing potential may have a detrimental effect by simultaneously stimulating the normal tissue to generate cytoprotective cues, creating niches that promote the survival of tumour cells [11,12,13]. Hence, the use of high throughput co-culture models composed of MM and stromal cells from various BM compartments is fundamental for a more precise understanding of the response to pharmacological compounds [13]. BM mesenchymal/fibroblastic cells comprise a well-accepted cytoprotective BM niche that inhibits drug efficacy against MM cells [6,13,14,15] that can be recapitulated by the BM stromal cell line HS5 in in vitro studies [14]. Based on the co-culture of MM cell lines with HS5 BM stromal cells [14], we developed an in vitro fluorescence-based high throughput platform that allows for the analysis of the concomitant effect of drugs on both MM and BM stromal cells [13]. The model is based on eGFP-expressing MM cell lines and mCherry-expressing HS5 cells that maintain the properties of the parental cell lines to study proliferation, survival, adhesion, and tracking of MM cells in co-culture with other BM cells. It also allows us to discriminate MM and stromal cell proliferation, as well as the separate analysis of MM and BM stromal cells using flow cytometry and imaging techniques.

Using this model, we found that, in the presence of MM cells, dexamethasone and bortezomib can paradoxically stimulate the proliferation and the development of a myofibroblastic phenotype in the BM mesenchymal cells, leading to MM drug resistance [13]. Fibroblasts contribute to drug resistance in MM by providing pro-survival and pro-angiogenic factors in the tumour microenvironment [15]. Additionally, fibroblasts can contribute to disease progression [15] by secreting particular extracellular matrix proteins [16] that assemble a fibrotic microenvironment in the BM associated with a poor prognosis of MM patients [17]. Our results suggest that, similarly to other cancers [11,12,13], the impact of MM therapies on the BM stroma may support drug resistance through the activation of the BM stroma, and this should be taken as a primary consideration in studies to determine the biology of MM tumours or drug efficacy.

Although the cytoprotection of MM cells by the BM mesenchymal/fibroblastic niche is usually considered in in vitro studies [6,13,14,15], there are other essential BM stromal cell types that are not commonly investigated. One significant example is the osteoclast (OC) niche, despite it being involved in disease progression in a large proportion of MM cases. Up to 80% of MM patients will develop bone disease at some point [18] due to increased OC activity and the repression of osteoblast function, induced by MM cells [6,18]. This results in the formation of lytic lesions and bone fractures, a major cause of morbidity in MM patients. Given that OCs can also provide cytoprotection to MM cells [19,20], we postulate that the OC niche should be considered routinely in studies to understand the mechanisms of drug resistance to current therapies and in drug discovery screenings.

Herein, we propose a sequence of analysis based on our high throughput platform for the rapid screening of possible compounds effective for targeting the cytoprotection mediated by the BM mesenchymal/fibroblastic niche and OCs. Using this model, we identified the signalling cascade downstream of PI3K Class IA proteins as a central pathway involved in MM resistance to dexamethasone and other current therapeutic drugs, such as thalidomide-derivatives (IMiDs) and bortezomib. Our results suggest that MM patients, in general, and particularly those with active bone disease, may benefit from combining the current therapeutic regimes with pan-PI3K Class IA inhibitors, such as GDC-0941, for improved outcomes.

## 2. Materials and Methods

### 2.1. Cell Lines

The Human eGFP-MM1.S, eGFP-MM1.R, and eGFP-RPMI8226 and mCherry-HS5 cells were previously generated using lentiviral vectors [13]. The original cell lines that were modified to express eGFP were obtained from the European Collection of Authenticated Cell Cultures (ECACC). The mouse cell line GFP-5T33 was kindly provided as a gift from Dr Manuchehr Abedi-Valugerdi from the Division of Hematology, Department of Medicine, Karolinska Institutet at Karolinska University Hospital, Stockholm, Sweden [21]. Cell lines were cultured at 37 °C in a humidified atmosphere in the presence of 5% CO_2,_ 95% air. Cells were cultured in RPMI-1640 medium, except for eGFP-RPMI 8226, mCherry-HS5 cells, and GFP-5T33 that were cultured in DMEM with L-glutamax. Culture media were supplemented with 10% foetal bovine serum (FBS). Cell lines were kept in culture for a maximum of eight passages to ensure the stability of the phenotype.

### 2.2. Determination of Cell Proliferation in eGFP-MM Cell Lines in Co-Culture with m-Cherry-HS5 Cells

m-Cherry-HS5 BM fibroblastic stromal cells were seeded at 5 × 10^3^ cells/per well in 96 well plates and incubated overnight in DMEM supplemented with 10% FCS. The following day, the culture media was aspirated and GFP positive MM cells were layered on m-Cherry-HS5 cells at a density of 2 × 10^5^ cells/mL in 200 μL per well of RPMI supplemented with 10% FCS. Three technical replicas were seeded per experimental condition. The library of compounds tested in our high throughput studies was Library I-384 from Merck. To determine the proliferation index, the fluorescence intensity (FI) per well was read at λex488 nm/λem528 nm and at λex584 nm/λem607 nm to detect cell numbers of eGFP-tumour cells and mCherry-HS5 cells, respectively, using a FLx800 multidetection microplate reader (Biotek Instruments, Winooski, VT, USA). Measurements were taken at d0 and d6 and the proliferation index was calculated as the ratio of the fluorescence emission at d6/d0 after subtracting the background emission. Each experiment was repeated three times.

### 2.3. Determination of MM Cell Viability in Co-Culture with OCs

Osteoclasts were differentiated for 21 days from primary BM or peripheral blood mononuclear cells obtained from MM patients, as previously described [22]. To determine MM cell viability, cultures were harvested and Annexin-V-APC staining in the eGFP positive population (MM cells) was measured by flow cytometry using a BD FACSCanto II flow cytometer (BD BioSciences, Franklin Lakes, NJ, USA) equipped with a high throughput sampler.

### 2.4. Cell Adhesion Assays

For the adhesion assays of MM cells on stromal cells, loosely adhered eGFP-expressing cells were removed by washing the wells twice with RPMI. The percentage of adhering tumour cells from the initial number of seeded cells was quantified by using the following equation: %adhesion = (Fl2 − Autofl1)/(Fl1 − Autofl2) × 100, where Fl1 and Fl2 correspond to the values of FI emitted by the fluorescent MM cells seeded into each well before and after washing, respectively; and Autofl1 and Autofl2 are the autofluorescence values from wells seeded with non-labeled tumour cells before and after the washing step, respectively.

### 2.5. Analysis of Cell Area and Formation of F-Actin Rings in OCs

Differentiated OCs cells were fixed for 20 min in 4% paraformaldehyde/3% sucrose in PBS warmed to 37 °C and permeabilized with 0.5% Triton X-100 in PBS for 10 min. After three washes with PBS, cells were then stained with Alexa 568–conjugated phalloidin (dilution 1:200) and with DAPI (dilution 1:500) to detect the distribution of F-actin and nuclei, respectively. After 1 h of incubation, coverslips were washed three times with distilled water and mounted using Vectashield ^®^ antifade mounting medium. Images were acquired using a Nikon Eclipse widefield fluorescence microscope. Exported TIFF images exported from the NIS-Elements AR 5.10.00 acquisition software were analysed to determine the OC area using FIJI software (https://imagej.net/software/fiji/downloads) (accessed on 1 February 2022). Twenty-five fields of view were quantified per experimental condition and the experiments were repeated three times.

### 2.6. In Vitro Bone Resorption Analysis

After 15 days of the in vitro differentiation of OCs on bone discs, cultures were left untreated or were treated with the corresponding test drugs for a subsequent 7 days, replenishing the cultures with fresh culture medium and drug treatments at day 5. At day 7, bone discs were removed from the wells, immersed in 10% NaOCl (BDH, Poole, UK) for 10 min to remove cells, washed and sonicated in distilled water for 10 min. After drying, discs were stained with a 1% crystal violet solution to enhance the contrast of bone resorption pits, and photographs were obtained by reflected light microscopy (objective 20×) in a number that would cover the entire substrate surface. Resorption trails and pits were analysed using FIJI. We quantified the percentage of the area occupied by resorption activity per field of view in three discs per experimental condition.

### 2.7. In Vivo Testing of Drug Efficacy Using the GFP-5T33 and C57BL/KaLwRij Mouse Model

Female and male C57BL/KaLwRij mice were purchased from Harlan Laboratories Ltd., UK and housed in the animal facilities at the Denmark Hill campus of King’s College London. All mice were 8 to 10 weeks old at the beginning of each experiment and they were housed under standard conditions. Food and water were provided ad libitum. All of the experiments were covered by the Home Office project licence number: PIL 70/06950. Mice were tail vein-injected with 2 × 10^5^ GFP-5T33 cells in 0.1 mL of PBS, and after the randomisation of injected mice in groups of 10 at day 20 after the injection, mice were left untreated or treated daily with dexamethasone (10 mg/Kg), or dexamethasone and GDC-0941 at 10 mg/Kg and 30 mg/Kg, respectively. Mice were daily weighed and monitored twice daily for signs of stress (hunching, piloerection, lethargy, difficulty moving, difficulty breathing, skin rash, pallor, diarrhoea) or paraplegia. Mice were humanly culled if they exhibited any one of the following: weight loss of 20% of the pre-treatment weight or difficulty breathing or difficulty moving or paraplegia. Additionally, mice exhibiting weight loss of the 15% pre-treatment weight and any one of the following that persisted for 48 h: hunching, piloerection, lethargy, skin rash, pallor, or diarrhoea were humanly culled.

### 2.8. Statistics

GraphPad Prism 9 software was used for statistical analysis using the adequate tests. The statistically significant difference using ANOVA, Student *t*-test, or Chi square, logrank test for the trend was determined from *p* < 0.05.

## 3. Results

### 3.1. PI3K Class IA Kinases Regulate a Signalling Node Involved in MM Drug Resistance to Dexamethasone Mediated by the BM Mesenchymal/Fibroblastic and OC Niches

MM patients are commonly treated with dexamethasone alone or in combination with new therapeutic drugs at presentation, as well as during relapse [1,3,23,24,25,26]. However, MM cells develop resistance to dexamethasone, and relapse is a widespread problem. Using our in vitro co-culture fluorescence-based platform, we have previously found that dexamethasone promotes BM stromal cell proliferation contributing to the cytoprotection of MM cells [13].

In the current study, we use the same high throughput experimental model to identify possible mechanisms of drug resistance to dexamethasone by screening a library of compounds that target signalling pathways known to be triggered by various growth factors and cell adhesion molecules. The GFP-MM1.S cell line was used in the initial high throughput steps of the screening, selected for being commonly used in MM studies [13,14] and sensitive to treatment with dexamethasone. GFP-MM1.S cells were cultured alone or in co-culture with mCherry-HS5 cells and were treated with 500 nM dexamethasone (half the maximum peak dose detectable in the plasma of treated patients [27]). Tested compounds used at 1 μM in combination with dexamethasone were selected as hits when they fulfilled all of the following criteria: (a) they reverted the mCherry-HS5 cells-mediated cytoprotection by decreasing the proliferation rate of co-cultured GFP-MM1.S cells treated with dexamethasone to equivalent or lower levels, as in MM cells treated in monoculture; (b) blocked MM cell adhesion on mCherry-HS5 cells; and (c) were inhibited by at least 15% of the dexamethasone-induced increase proliferation of mCherry-HS5 cells (Figure S1). A large number of compounds fulfilled the first and second criteria, but only 10% of them also achieved the inhibition of the dexamethasone-induced proliferation of mCherry-HS5 cells (Figure S1).

**Figure 1:**
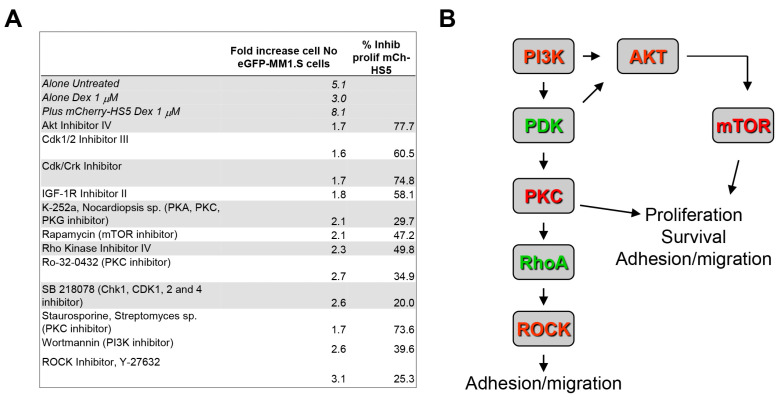
PI3K Class IA controls a signalling node involved in BM stroma-mediated drug resistance. (**A**) List of compounds selected in a high throughput screening for inhibiting cytoprotection of eGFP-MM1.S (dexamethasone sensitive) cells against dexamethasone (1 μM) provided by the BM stromal cell line HS5. Compounds were selected for their significant inhibition of MM cell adhesion on stromal cells, as well as of dexamethasone-induced proliferation of HS5 stromal cells by at least 15%. These compounds also blocked OC-mediated cytoprotection of GFP-MM1.S cells against dexamethasone-induced apoptosis. All of the experiments were performed in triplicate and three technical replicas per experimental condition were analysed in each experiment; (**B**) diagram illustrating the related signalling pathways identified as targets by the selected compounds from the Merck Library listed in (**A**) and their roles in the regulation of cell functions. The red colour indicates the direct targets of the compounds used, the green colour the targets inhibited as a consequence of the upstream-inhibited kinases.

The same high throughput screening was also performed on GFP-MM1.S cells co-cultured with OCs (Figure S2). We have previously shown that the observed decreased proliferation of MM cells in the presence of OCs when treated with pharmacological compounds is commonly caused by a cytostatic rather than a cytotoxic effect induced by OC-mediated cytoprotection [13]. Then, compounds that may inhibit the cytoprotection against dexamethasone mediated by OCs were selected on their basis for their capacity to significantly increase the percentage of apoptotic cells by at least 25% above the viability of untreated MM cells. We identified in our drug screening some compounds that prevented cytoprotection by OCs or mCherry-HS5 cells but failed to prevent the cytoprotection by both cell types. These included inhibitors of the EGF receptor, CDK4, Bcr-abl, PDGF receptor, and NF-κB (Figure S2). Twelve compounds significantly inhibited cytoprotection mediated by both the BM mesenchymal/fibroblastic cell line mCherry-HS5 and OCs (Figure 1A). These included inhibitors of CDK1 and the IGF1 receptor, as well as inhibitors of the PI3K/AKT/mTOR signalling pathway, of PKC, and of the RhoA/ROCK pathway. Interestingly, many of these pathways are interconnected and promote cell survival, proliferation, adhesion, and migration downstream of PI3K (Figure 1B).

Taken together, our results indicate that the interrelation between the PI3K/AKT/mTOR and the PI3K/PKC/RhoA pathways may contribute to the drug resistance of MM cells against dexamethasone mediated by the BM mesenchymal stromal and OC niches. Since PI3K is a vital regulator upstream of both pathways, these results prompted us to test the possible efficacy of PI3K inhibitors to overcome BM stroma mediated cytoprotection against dexamethasone.

### 3.2. Inhibition of Single PI3K Class IA Isoforms Fails to Prevent Resistance to Dexamethasone Mediated by BM Mesenchymal Cells and OCs

Several previous studies have investigated the use of pharmacological inhibitors that would directly target the PI3K class IA pathway for their efficacy against MM. Two previous studies reported that targeting the PI3K class I isoforms α [28] and δ [29] showed efficacy in vitro to overcome BM mediated drug resistance, mediated by the mesenchymal/fibroblastic niche. However, these studies did not address the possible capacity of these compounds for the therapy-induced activation and proliferation of cytoprotective BM stromal cells, or the possible cytoprotection by the OC niche.

We found that inhibitors of PI3Kα and γ isoforms were the only efficacious drugs as single agents and inhibited eGFP-MM1.S cell proliferation in the presence of cytoprotective BM mesenchymal/fibroblastic mCherry-HS5 cells (Figure S3). Blocking the activity of PI3Kα enhanced the efficacy of dexamethasone to repress eGFP-MM1.S cell proliferation when cells were cultured alone or in the presence of cytoprotective mCherry-HS5 cells (Figure S3A). It also inhibited MM cell adhesion on stromal cells, but it failed to effectively prevent the dexamethasone-enhanced proliferation of mCherry-HS5 cells (Figure S3A–C), which may contribute to the observed remaining cytoprotection observed in combination with dexamethasone (Figure S3A). In the presence of mCherry-HS5 cells, blocking PI3Kγ failed to increase the efficacy of dexamethasone with the same efficiency as PI3Kα, correlating with the lack of impact on the adhesion of eGFP-MM1.S or on the dexamethasone-mediated increased proliferation of mCherry-HS5 cells (Figure S3J–L). The inhibition of the PI3K β or δ isoforms as single agents or in combination with dexamethasone completely failed to prevent MM cell proliferation (Figure S3D–I).

We then analysed the involvement of the specific PI3K isoforms in the cytoprotection mediated by the OC niche. The inhibition of the PI3Kα, and particularly of the PI3Kβ isoforms, significantly reduced, albeit partially, the OC-mediated cytoprotection against dexamethasone (Figure S4A,B). The inhibition of PI3K δ or γ did not show any cytotoxic effect or increased dexamethasone efficacy in the presence of OCs (Figure S4C,D).

Taken together, our results support previous reports indicating that the PI3Kα is the predominant isoform involved in MM survival and the interaction with the BM mesenchymal/fibroblastic niche [28] that contributes to cytoprotection to current therapeutic drugs. However, our data also suggest that the inhibition of this isoform fails to block the expansion of the cytoprotective BM mesenchymal niche promoted by MM cells in response to drug treatments, which could contribute to the drug resistance to these agents [13,15]. Additionally, in the OC co-culture setting, although the inhibition of the PI3Kα may enhance the efficacy of dexamethasone, this isoform is not effective for targeting the bone resorptive activity of OCs, which is dependent on the PI3Kβ and δ isoforms [30]. Overall, our results suggest that targeting single PI3K class IA isoforms in isolation may be a limited approach for targeting the overall cytoprotection mediated by various cell types of the BM stroma concomitantly. We then investigated whether targeting all isoforms simultaneously may prove a more efficient strategy to prevent resistance to current MM treatments, in comparison to inhibiting particular isoforms in isolation.

### 3.3. Simultaneous Targeting of All PI3K Class IA Isoforms with GDC-0941 Overcomes Resistance to Dexamethasone Mediated by Both BM Mesenchymal Cells and OCs

We studied the efficacy of the pan-PI3K Class IA inhibitor GDC-0941 to overcome resistance to dexamethasone. This compound has already been shown to have some pharmacological activity against MM in in vitro and in vivo studies [31]. However, tumour cells can develop tumour microenvironment-mediated resistance to GDC0941 [32,33], and the previous studies in MM lack a systematic analysis of cytoprotection mediated by the OC-niche. Additionally, previous studies in MM did not address the impact of GDC-0941 alone or in combination with currently used therapeutic drugs on the proliferation of BM mesenchymal/fibroblastic cells.

**Figure 2:**
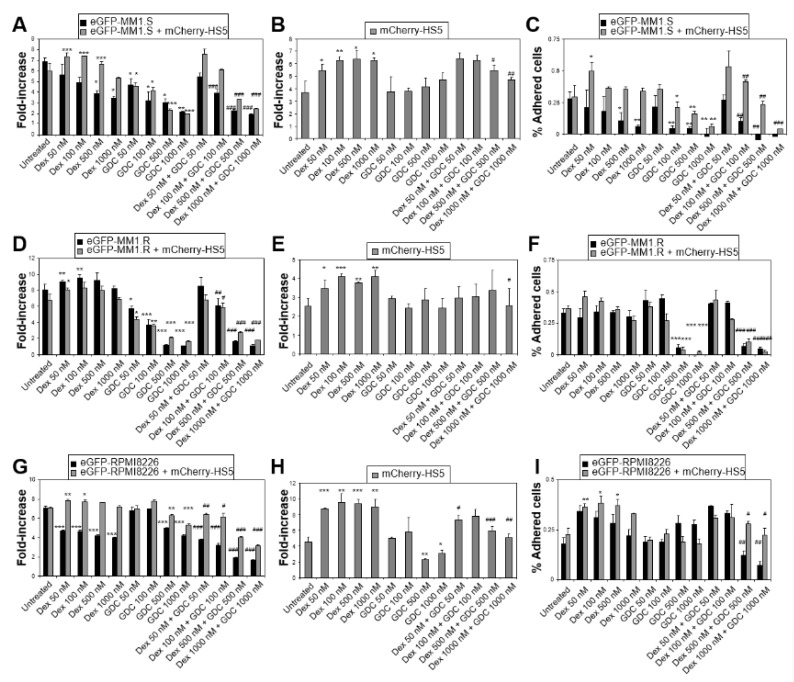
GDC-0941 inhibits BM mesenchymal stromal cell-mediated cytoprotection of MM cells against dexamethasone. Fluorescent-based analysis of proliferation of eGFP-MM1.S, eGFP-MM1.R, and eGFP-RPMI8226 cells (**A**, **D,** and **G**, respectively) cultured alone or in the presence of mCherry-HS5 cells and left untreated or treated with dexamethasone (dex) or GDC0941 (GDC) as single agents or in combination for 6 days and analysed by fluorimetry. Bar charts show the average and the SD of fold increase of proliferation; (**B**,**E**,**H**) fluorescent-based analysis of proliferation of mCherry-expressing HS5 stromal cells with MM cells of eGFP-MM1.S, eGFP-MM1.R, respectively; (**C**,**F**,**I**) percentage of adhered eGFP-MM1.S, eGFP-MM1.R, and eGFP-RPMI8226 cells on mCherry-HS5 cells, respectively. * *p* < 0.05; ** *p* < 0.005; *** *p* < 0.001 with respect to untreated under the same experimental condition (alone or in co-culture with mCherry-HS5; # *p* < 0.05; ## *p* < 0.005; ### *p* < 0.001 with respect to Dex treatment, two-way ANOVA test.

At doses achievable in patients [34], GDC-0941 as a single agent inhibited the proliferation of the cell lines eGFP-MM1.S and eGFP-MM1.R (dexamethasone sensitive and resistant, respectively), and synergised with dexamethasone blocking the cytoprotection mediated by mCherry-HS5 cells (Figure 2A–F). This correlated with the inhibition of the adhesion of MM cells on the stroma, as well as the blockade of dexamethasone-induced mCherry-HS5 cell proliferation. However, GDC-0941 as a single agent showed a lower efficacy to inhibit the proliferation of the cell line GFP-RPMI8226 in comparison to the effect on eGFP-MM1.S and eGFP-MM1.R cells (Figure 2G,I). This correlated with the previously reported lower level of activation of the PI3K pathway in RPMI8226 cells vs. MM1.R cells [31]. Additionally, GFP-RPMI822 cells showed resistance to GDC-0941 as a single agent mediated by mCherry-HS5 cells, suggesting some kind of stromal-mediated mechanism of protection of these MM cells. Nevertheless, GDC-0941 inhibited the BM stromal-mediated resistance of GFP-RPMI822 cells to treatment with dexamethasone, correlating with the inhibition of the proliferation of mCherry-HS5 cells (Figure 2H).

**Figure 3:**
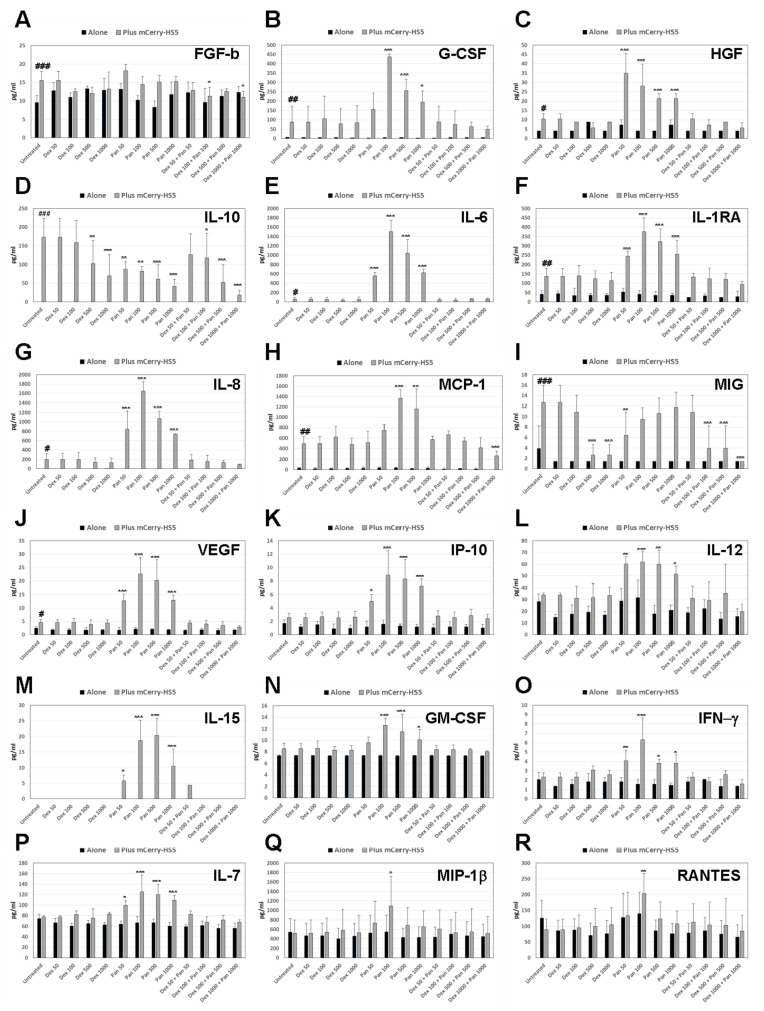
Secretion of cytokines in response to drug treatments in cultures of GFP-RPMI8226 MM cells cultured alone or in the presence of the BM mesenchymal stromal cell line mCherry-HS5. High throughput analysis of cytokine secretion in the supernatants of cultured cells left untreated or treated with dexamethasone (dex) or GDC-0941 (PAN) as single agents or in combination for 2 days. Supernatants were collected and subjected to multiplex cytokine analysis to determine the concentration of FGF-b (**A**), G-CSF (**B**), HGF (**C**), IL-10 (**D**), IL-6 (**E**), IL-1RA (**F**), IL-8 (**G**), MCP-1 (**H**), MIG (**I**), VEGF (**J**), IP-10 (**K**), IL-12 (**L**), IL-15 (**M**), GM-CSF (**N**), IFN-γ (**O**), IL-7 (**P**), MIP-1β (**Q**) and RANTES (**R**). Bar charts show the average and the SD of the concentration of each cytokine detected in the supernatants in pg/mL. * *p* < 0.05; ** *p* < 0.005; *** *p* < 0.001 with respect to untreated under the same experimental condition (alone or in co-culture with mCherry-HS5; # *p* < 0.05; ## *p* < 0.005; ### *p* < 0.001 with respect to Dex treatment, two-way ANOVA test.

We then investigated possible mechanisms of stroma mediated cytoprotection against GDC-0941 as a single agent. The levels of secretion of soluble factors by MM and stromal cells largely influences the overall response to treatments [35,36]. A high throughput analysis of the cytokine secretion of GFP-RPMI8226 cells showed that the co-culture of MM with mCherry-HS5 cells resulted in the increased secretion of critical cytokines known to contribute to stroma-mediated drug resistance: IL-6, FGF-b, G-CSF, HGF, IL-10, IL-1RA, IL-8, MCP-1, MIG, and VEGF (Figure 3). These cytokines and growth factors are known to contribute to different aspects of tumour progression, including cell survival and proliferation (IL-6), fibroblast proliferation, and the generation of a fibrotic microenvironment (FGF-b), angiogenesis (VEGF), MM invasive migration (IL-6, HGF), recruitment and the differentiation of leukocytes to the tumour microenvironment (G-CSF, IL-8, MCP-1, MIG), and immunosuppression (IL-100 and IL-1RA). Treatment with dexamethasone as a single agent did not prevent the secretion of these factors, except when used at the highest doses achievable in patients [27], which inhibited the secretion of IL-10 and MIG (Figure 3D,I). The treatment of co-cultures with GDC-0941 as a single agent more than doubled the secretion of all of the cytokines under study, except for MIP-1b, RANTES, and FGF-b. Only IL-10 secretion was reduced by treatment with GDC-0941 (Figure 3D). This unwanted increase in the secretion of pro-tumoural growth factors in response to GDC-0941 as a single agent may contribute to the resistance of some MM cells, such as eGFP-RPMI 8226 in the presence of BM mesenchymal/fibroblastic cells, and may represent a process involved in the previously observed resistance to this agent [32,33]. The combination of dexamethasone with GDC-0941 blocked the undesired increased secretion of cytokines induced by the stromal cells treated with GDC-0941 as a single agent (Figure 3).

**Figure 4:**
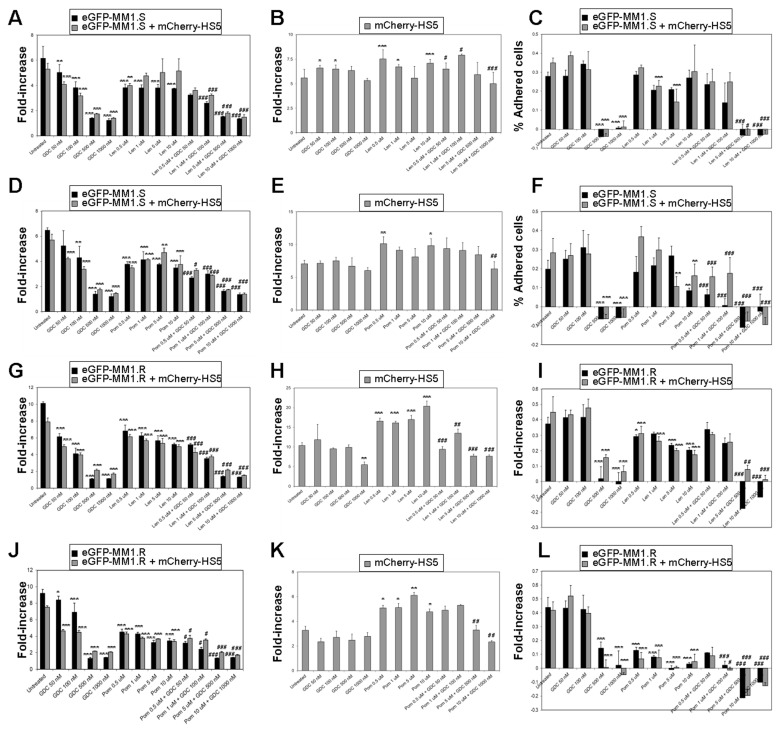
GDC-0941 inhibits BM mesenchymal stromal cell-mediated cytoprotection of MM cells against lenalidomide and pomalidomide. Fluorescent-based analysis of proliferation of eGFP-MM1.S (**A**,**D**) and eGFP-MM1.R cells (**G**,**J**) cultured alone or in the presence of mCherry-HS5 cells and left untreated or treated with dexamethasone (dex) or lenalidomide (Len) or pomalidomide (Pom), as single agents or in combination of lenalidomide or pomalidomide with dexamethasone for 6 days and analysed by fluorimetry. Bar charts show the average and the SD of fold increase of proliferation; (**B**,**E**,**H**,**K**) fluorescent-based analysis of proliferation of mCherry-expressing HS5 stromal cells with MM cells eGFP-MM1.S (**B**,**E**) or eGFP-MM1.R (**H**,**K**); (**C**,**F**,**I**,**L**) percentage of adhered eGFP-MM1.S (**C**,**F**) and eGFP-MM1.R (**I**,**L**) cells on mCherry-HS5 cells, respectively. * *p* < 0.05; ** *p* < 0.005; *** *p* < 0.001 with respect to untreated under the same experimental condition (alone or in co-culture with mCherry-HS5); # *p* < 0.05; ## *p* < 0.005; ### *p* < 0.001 with respect to single treatment of Len or Pom, two-way ANOVA test.

GDC-0941 also enhanced the efficacy of IMiDs (lenalidomide and pomalidomide) (Figure 4). Using eGFP-MM1.S and eGFP-MM1.R cells, we found that mCherry-HS5 cells offered no or minimal cytoprotection against lenalidomide or pomalidomide, and the MM killing capacity of these compounds was significantly enhanced when combined with GDC-0941. Co-treatment resulted in the increased inhibition of MM cell adhesion on stromal cells (Figure 4C,F,I,L) and the repression of the increase in the proliferation rate of BM mesenchymal/fibroblastic stromal cells induced by IMiDs (Figure 4E,H,K). Overall, our results suggest that GDC-0941 may be most efficacious when used in combination with dexamethasone, as well as other therapeutic drugs, rather than as a single agent.

**Figure 5:**
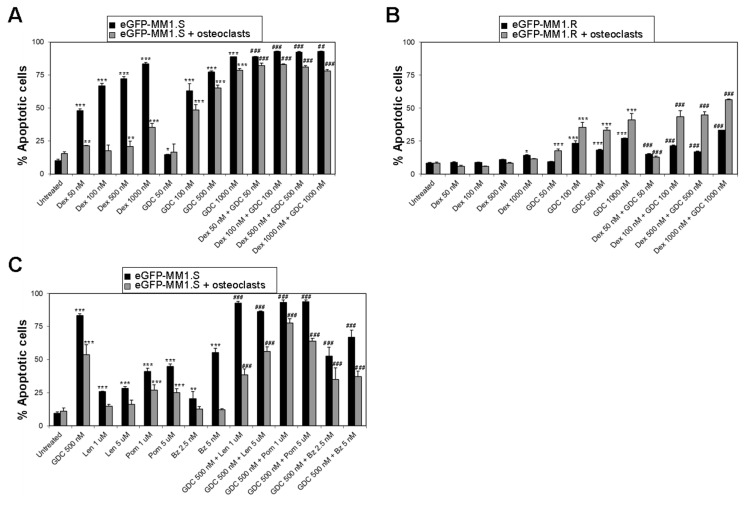
GDC-0941 inhibits the osteoclast-mediated protection of MM cells against dexamethasone. (**A**,**B**) Bar charts show the average percentage of apoptotic cells (Annexin V positive) ± SD in cultures of eGFP-MM1.S (**A**) and eGFP-MM1.R cells (**B**) untreated or treated for 3 days with dexamethasone (dex) or GDC-0941 (GDC) as single agents or in combination, cultured alone or in the presence of Ocs; (**C**) analysis of the efficacy of GDC0941 to overcome the anti-apoptotic effect of OCs against treatment with lenalidomide (Len), pomalidomide (Pom), or bortezomib (Bz). * *p* < 0.05; ** *p* < 0.005; *** *p* < 0.001 with respect to untreated under the same experimental condition (alone or in co-culture with mCherry-HS5; # *p* < 0.05; ## *p* < 0.005; ### *p* < 0.001 with respect to Len, Pom, or Bz as single treatments, two-way ANOVA test.

We then investigated the efficacy of GDC-0941 to overcome the cytoprotection of MM cells against dexamethasone mediated by the OC niche. The presence of OCs resulted in high levels of cytoprotection of eGFP-MM1.S cells against dexamethasone and GDC-0941 when used as single agents (Figure 5A). However, the combination with GDC-0941 significantly enhanced the efficacy of dexamethasone in the presence of OCs. The eGFP-MM1.R cell line responded to GDC-0941 as a single agent, independently of the presence of OCs but this effect was also enhanced when combined with dexamethasone (Figure 5B). These results suggest that GDC-0941 can overcome the cytoprotection of MM cells against dexamethasone mediated by OCs, even if MM cells develop autonomous mechanisms of resistance to this drug. We then tested whether GDC-0941 improved the efficacy in the OC niche of other therapeutic drugs. The co-culture of eGFP-MM1.S cells with OCs also resulted in cytoprotection against lenalidomide, pomalidomide, and bortezomib as single agents (Figure 5C) and GDC-0941 significantly increased the pro-apoptotic effect of these drugs in the presence of OCs.

Taken together, our results indicate that simultaneously targeting all of the PI3K class IA isoforms with GDC-0941 effectively inhibits BM mesenchymal/fibroblastic cells and the OCs-mediated cytoprotection of MM cells against dexamethasone and other therapeutic drugs.

### 3.4. GDC-0941 Inhibits Bone Resorption by Osteoclasts by Repressing OC Differentiation and the Organisation of Sealing Zones

**Figure 6:**
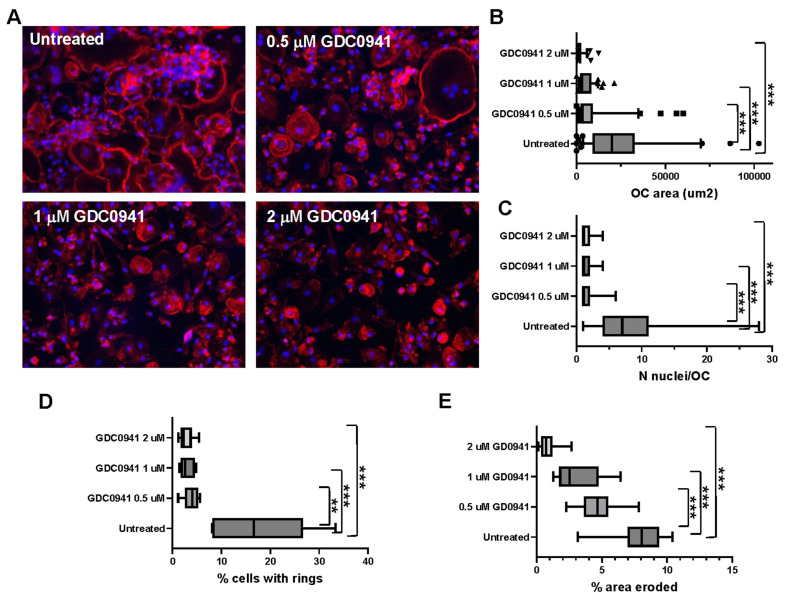
GDC0941 inhibits osteoclasts differentiation, cytoskeletal organisation, and bone resorption. (**A**–**D**) Osteoclasts derived from peripheral blood from multiple myeloma patients and cultured on glass for 2 weeks were treated with dexamethasone alone or in combination with GDC-0941. (**A**) Micrographs showing the distribution of F-actin (red) and nuclei (blue). Magnification 20X lens; (**B**) quantification of the OC area of spreading in mm^2^; (**C**) quantification of the number of nuclei per OC; (**D**) percentage of the number of OC per field of view, ** *p* < 0.01; (**E**) similarly, OCs were derived on dentine discs. Cells were then lysed, and dentine discs washed and analysed for pit formation to determine percentage of bone erosion area. Box and whiskers diagrams of percentage of area eroded on the dentine disc. The experiments were performed in triplicate (three discs per condition) and repeated twice. Significant differences were observed at *** *p* < 0.005 with respect to untreated cells as indicated (Kruskal Wallis test).

The majority of MM patients will present active bone disease during disease progression, making it crucial that efficacious therapies prevent cytoprotection of MM cells by OCs and the formation of lytic lesions. We have previously shown that dexamethasone as a single agent does not have an impact on OC differentiation or bone resorption activity [13]. All of the proteins that are part of the PI3K-regulated node involved in MM resistance, identified in our drug screening (Figure 1), are also regulators of the cytoskeletal organisation of osteoclasts required for effective bone resorption [30,37,38,39,40]. We then tested whether GDC-0941 prevented the bone resorptive activity of OC. Untreated OCs were multinucleated with well-developed F-actin rings (the structure used by OCs to seal the membrane onto the bone to release enzymes locally for bone resorption) (Figure 6). Treatment with GDC-0941 resulted in a concentration-dependent decrease in the area of spreading (Figure 6A,B) and in the number of nuclei per OC (Figure 6A,C), as well as in the repression of the formation of F-actin rings (Figure 6A,D), indicating defects in OC differentiation and the organisation of F-actin. As a consequence, treatment with GDC-0941 inhibited the in vitro bone resorption of OC (Figure 6E).

### 3.5. GDC-0941 Enhances the In Vivo Efficacy of Dexamethasone in the GFP-5T33/C57Rawji MM Mouse Model

**Figure 7:**
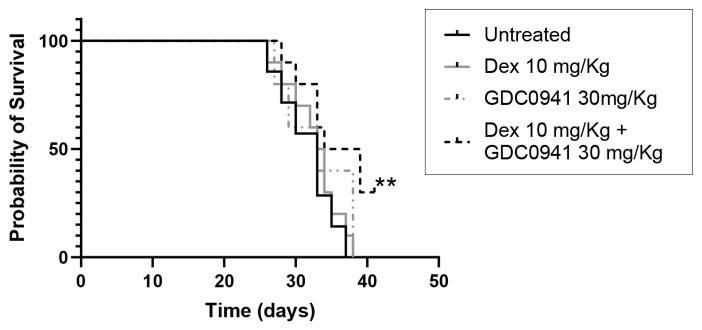
Dexamethasone in combination with GDC0941 increases survival of C57BL/KaLwRij mice inoculated with eGFP-5T33 MM cells. Pattern of survival of C57BL/KaLwRij mice inoculated with 2 × 10^5^ eGFP-5T33 MM cells via the tail vein from the time of initiation of drug treatment (15 days post-inoculation). Mice were left untreated or treated with 10 mg/Kg dexamethasone (Dex) or 30 mg/Kg GDC-0941 as single agents or in combination. Treatment with dexamethasone or GDC-0941 as single agent failed to improve mice survival. Combination of 10 mg/Kg dexamethasone with 30 mg/Kg GDC-0941 significantly increased mice survival. Significant differences were observed at ** *p* < 0.01 with respect to untreated (Chi square, logrank test for trend).

A previous study assessed the efficacy of GDC-0941 alone or in combination with clinical drugs against MM, using an in vivo model comprised of subcutaneous xenografts of MM cell lines in immunosuppressed mice [31]. Although this model is valid as an initial step to investigate the in vivo efficacy of drugs against MM, it is limited by the absence of the BM microenvironment in the subcutaneous MM tumours developed in these mice. Our current data indicate a critical role of the BM niches in the possible mechanisms of action of GDC-0941 to prevent cytoprotection against dexamethasone. We then decided to use the immunocompetent syngeneic mouse model in our study, composed of eGFP-5T33 cells injected in C57BL/KaLwRij mice and colonizing the BM [41], which is a highly representative model of disseminated MM. Treatment with dexamethasone at 10 mg/Kg or GDC0941 at 30 mg/Kg as single agents did not increase the survival of mice bearing MM tumours (Figure 7). However, when combined, mice survival significantly increased, with 30% of animals remaining alive for at least 3 days after all untreated or single agent-treated mice had already died from MM disease. Our in vivo results support the ability of GDC-0941 to increase the efficacy of dexamethasone against MM tumours.

## 4. Discussion

During disease progression, MM cells commonly activate OCs, promoting their bone resorptive activity and leading to devastating lytic lesions that result in pain, bone fractures, and restricted mobility of MM patients. Activated OCs also release growth factors and cytokines that contribute to tumour development by promoting MM cell survival and proliferation [19,42,43,44], as well as the further activation of other BM cytoprotective niches, such as BM mesenchymal/fibroblastic stromal [42] and endothelial cells [45]. In turn, MM cells and the activated BM stromal compartments release osteoclastogenic factors [46], resulting in a vicious feedback loop of MM tumour growth and bone damage [19,42]. Despite the key role of osteoclasts in the cytoprotection of MM cells against drug treatments [19,42], the contribution of this BM niche is not commonly evaluated in pre-clinical studies to determine the efficacy of drugs to overcome tumour microenvironment mediated drug resistance. The great majority of studies determine the potential to develop BM-mediated MM drug resistance in MM cells co-cultured in the presence of BM fibroblastic/mesenchymal cells. These stromal cells can be derived from BM aspirates from MM patients and very commonly, the BM stromal cell line HS5 is used as a substitute instead of primary cells, as HS5 cells very closely recapitulate the development of some critical MM resistant traits detected in MM patients [14]. Our current report indicates that valuable studies should also include experiments considering the cytoprotection of MM cells by OCs. This idea was previously proposed more than a decade ago when the essential contribution of the OC niche to resistance to therapy in MM became clearly apparent [19]. Perhaps the convenience of using HS5 cells, which are easier to culture than primary cells (they are commercially available and do not require supplementation with complex and costly reagents) has relegated the use of co-cultures of MM cells with OCs in preclinical studies.

However, we show herein that OCs derived from peripheral blood mononuclear cells from MM patients or healthy individuals can be easily cultured in a high throughput setting using 96 well plates, and provide distinctive cytoprotection to MM cells against currently used clinical drugs. The use of eGFP-expressing MM cell lines [13] enables the use of this co-culture setting for an easy drug screening of hundreds of compounds in a short period of time. Using our straightforward analysis methodology of the impact of candidate drug compounds on co-cultures of MM cells with BM mesenchymal/fibroblastic cells vs. OCs, we have been able to demonstrate that there are pathways that may be involved in MM resistance to current therapeutic agents, such as dexamethasone, that are particular for each BM niche. For example, our data show that the signalling pathway regulated by NF-κB promotes resistance to dexamethasone mediated by BM mesenchymal/fibroblastic stromal cells, but not by OCs. The mechanisms of action of IMiDs and proteasome inhibitors involves the inhibition of the NF-κB signalling, which is thought to contribute to the enhanced efficacy of these compounds in the presence of BM cytoprotective stroma in comparison to previously used chemotherapeutic drugs [47]. IMiDs and proteasome inhibitors have also been shown to block the lytic activity of OCs by interfering with NF-κB signalling during OC maturation, resulting in reduced bone disease in patients [48,49,50]. Our data confirmed that IMiDs prevent the cytoprotection mediated by BM mesenchymal/fibroblastic cells. However, we also revealed that despite the inhibition of OC maturation and activity, IMiDs and bortezomib-treated OCs can still provide cytoprotection for MM cells. Similarly, we found that OCs protect MM cells against treatment with dexamethasone and this process is not dependent on the activity of NF-κB. These results indicate that in in vitro studies, in addition to determining the inhibitory capacity of new compounds to repress cytoprotection by BM mesenchymal cells and to block bone resorption by OCs, verifying their efficacy to block OC-mediated drug resistance must be routinely tested.

It has been previously shown that MM plasma cells alter the BM microenvironment by stimulating the proliferation of mesenchymal stromal cells [46] and our published and current data indicate that this process can be enhanced by drug treatments. We have previously shown that dexamethasone and bortezomib induce a proliferative and fibroblastic phenotype of BM mesenchymal cells in the presence of MM cells that contributes to the inhibition of drug efficacy [13]. The undesirable stimulation of cytoprotective niches by therapeutic drugs has also been described as a process involved in the development of drug resistance in various types of cancers [11,12]. Herein we show that, similarly, IMiDs can induce the expansion of co-cultured mCherry-HS5 cells in the presence of some MM cell lines. This stimulation of the expansion of fibroblastic stromal cells could contribute to the observed fibrotic microenvironment observed in MM tumours, which correlates with poor disease prognosis and drug resistance [15,17]. Hence, we propose that in preclinical studies, monitoring the impact of potential drug candidates on the proliferation and fibroblastic differentiation of BM mesenchymal cells in co-culture with MM cells, as described in our model, is vital to determine more accurately possible drug efficacy against MM. For example, BM-mediated resistance to pomalidomide involves the activation of ERK signalling, as determined by changes in the proliferation of MM cells grown in co-culture with primary BM stromal cells or their conditioned media [51]. Similarly to previous reports where they use MM cell proliferation as a single readout for drug efficacy, our current results also predict that ERK inhibitors could inhibit resistance to dexamethasone. However, we also found that the inhibition of ERK failed to block the dexamethasone-induced proliferation of BM mesenchymal stromal cells and it was inefficient to block OC-mediated drug resistance. Consequently, we rejected ERK as a possible target to overcome drug resistance in MM and prioritised other signalling pathways as possible targets.

Our results determined that BM-mediated resistance to dexamethasone can be inhibited by compounds that target the PI3K/Akt/mTOR signalling pathway, inhibitors of PKC and RhoA/ROCK inhibitors. All these pathways are interlinked and become activated downstream of PI3K Class IA. Previous studies have proposed the use of Akt inhibitors to target the PI3K/Akt/mTOR pathway in MM [52,53]. However, the PI3K pathway bifurcates, and the activation of PDK1 by PI3K is independent of Akt activation. Then, the inhibition of Akt may not result in a complete inactivation of the PI3K pathways and this therapeutic approach may fail [54]. Hence, we investigated whether directly targeting PI3K as the critical regulator upstream of the identified signalling node would demonstrate an improved strategy for MM cell killing to overcome resistance to dexamethasone.

Since several therapeutic agents targeting various isoforms of PI3K class IA proteins have previously been proposed for efficacy against MM [28,29,43], we tested them using our co-culture models. Our data show that inhibiting PI3Kα, as previously proposed [28], and γ isoforms reduced the survival of MM cells treated with dexamethasone in the presence of BM mesenchymal/fibroblastic stromal cells. However, the inhibition of these isoforms failed to efficiently block the dexamethasone-induced proliferation of mCherry-HS5 cells. Additionally, the most effective isoform that inhibited OC mediated drug resistance was PI3K β, which is also the critical isoform for the bone lytic activity of OCs [30]. Taken together, our data suggest that using inhibitors to individual PI3K isoforms may not efficiently overcome all the aspects from the BM niches involved in the cytoprotection of MM cells. We considered that a pan-inhibitor of the PI3K Class IA isoforms would provide the most efficacious approach to repress the identified mechanisms of drug resistance. We then tested the MM cell killing efficacy of GDC-0941, an inhibitor with high activity against PI3Kα and PI3Kδ, as well as specific to the β and γ isoforms, albeit with a lower activity.

GDC-0941 inhibited the BM mesenchymal/fibroblastic and OC-mediated drug resistance of MM cells to current MM therapeutic drugs, as well as the bone lytic activity of osteoclasts. The secretion of protons by OCs to activate the activity of bone degradative enzymes can promote tumour expansion [44] involving the activation of the PI3K pathway in MM cells contributing to resistance [43], a process that would also be targeted by GDC-0941. GDC-0941 also prevented the expansion of BM-mesenchymal cells treated with dexamethasone, bortezomib, or IMiDs in the presence of MM cells, which may be explained by a direct effect of GDC-0941 on the activity of PI3K in BM stromal cells. Our results are in line with a very recent report showing that BM mesenchymal cells from MM patients with active disease express higher levels of gene transcription and protein expression of the genes involved in the PI3K/AKT/mTOR pathway. This correlates with the enhanced proliferation of BM mesenchymal cells induced by MM cells, which was also shown to be inhibited by GDC-0941 [55].

GDC-0941 has been shown to be well tolerated by patients, including MM [56], and several clinical trials are ongoing to test its anti-cancer activity as a single agent in various solid tumours. Our data suggest that clinical trials in MM should consider the toxicity and efficacy of GDC-0941 in combination with dexamethasone or some of the other current MM therapies for the best efficacy [57]. Our in vitro data indicate that when used in isolation, GDC-0941 may trigger the unwanted secretion of cytokines in the tumour microenvironment that could contribute to drug resistance. In our in vitro models, this process was prevented by combining GDC-0941 with dexamethasone. Additionally, targeting the PI3K/AKT/mTOR pathway in combinational therapies may allow for the use of lower doses of drugs, reducing the toxicity of current treatments, such as IMiDs [58].

Given the plasticity and heterogeneity of MM tumours [6,59], further high throughput studies using libraries of new compounds or using genetic ablation should be tested using our model for the rapid identification of potentially effective tailored and/or universal new therapies to prevent resistance and relapse. For example, targeting TAK1-PIM2 signalling has also been reported to inhibit cytoprotection mediated by BM mesenchymal cells and OCs [31].

## 5. Conclusions

In summary, our results show that our high throughput method and analysis comprises a more accurate approach for studying the cytoprotection of MM cells by the BM microenvironment by measuring the activity of MM and BM stromal cells in the mesenchymal and osteoclast niches. This is a vital approach for studying MM, as the overall response to drug treatments is dependent on their impact on both the MM plasma cell and BM stromal compartments. We also show that targeting PI3K Class IA kinases in MM tumours in combination with current therapeutic drugs [31], such as dexamethasone, may prevent drug resistance mediated by the mesenchymal and osteoclast niches, improving patient outcomes. These treatments may particularly benefit MM patients presenting bone disease.

## Data Availability

The data presented in this study are available in this article and supplementary material.

## Supplementary Materials

The following supporting information can be downloaded at: https://www.mdpi.com/article/10.3390/cancers15020462/s1, Figure S1: Analysis of the efficacy of compounds in combination with dexamethasone (dex) to overcome BM-mesenchymal cell cytoprotection of MM cells against dexamethasone, Figure S2: Analysis of the efficacy of compounds in combination with dexamethasone (dex) to overcome OC-mediated cytoprotection of MM cells against dexamethasone, Figure S3: Analysis of the efficacy of inhibiting individual PI3K Class I isoforms to overcome BM mesenchymal cell-mediated cytoprotecion of MM cells against dexamethasone, Figure S4: Analysis of the efficacy of inhibiting individual PI3K Class I isoforms to overcome OC-mediated cytoprotecion of MM cells against dexamethasone.
