# Supplementary material for: Inhibition of PI3K Class IA Kinases Using GDC-0941 Overcomes Cytoprotection of Multiple Myeloma Cells in the Osteoclastic Bone Marrow Microenvironment Enhancing the Efficacy of Current Clinical Therapeutics
Source: Cancers (Basel). 2023 Jan 11;15(2):462. doi: 10.3390/cancers15020462 (PMC9856454; doi:10.3390/cancers15020462)
Supplement: Supplementary file 1 [file cancers-15-00462-s001.zip › cancers-2077543-supplementary.pdf]

cancers

Supplementary Materials: Inhibition of PI3K Class IA Kinases Using GDC-0941 Overcomes Cytoprotection of Multiple Myeloma Cells in the Osteoclastic Bone Marrow Microenvironment Enhancing the Efficacy of Current Clinical Therapeutics

Hugh Kikuchi, Eunice Amofa, Maeve Mcenery, Steve Arthur Schey, Karthik Ramasamy, Farzin Farzaneh and Yolanda Calle

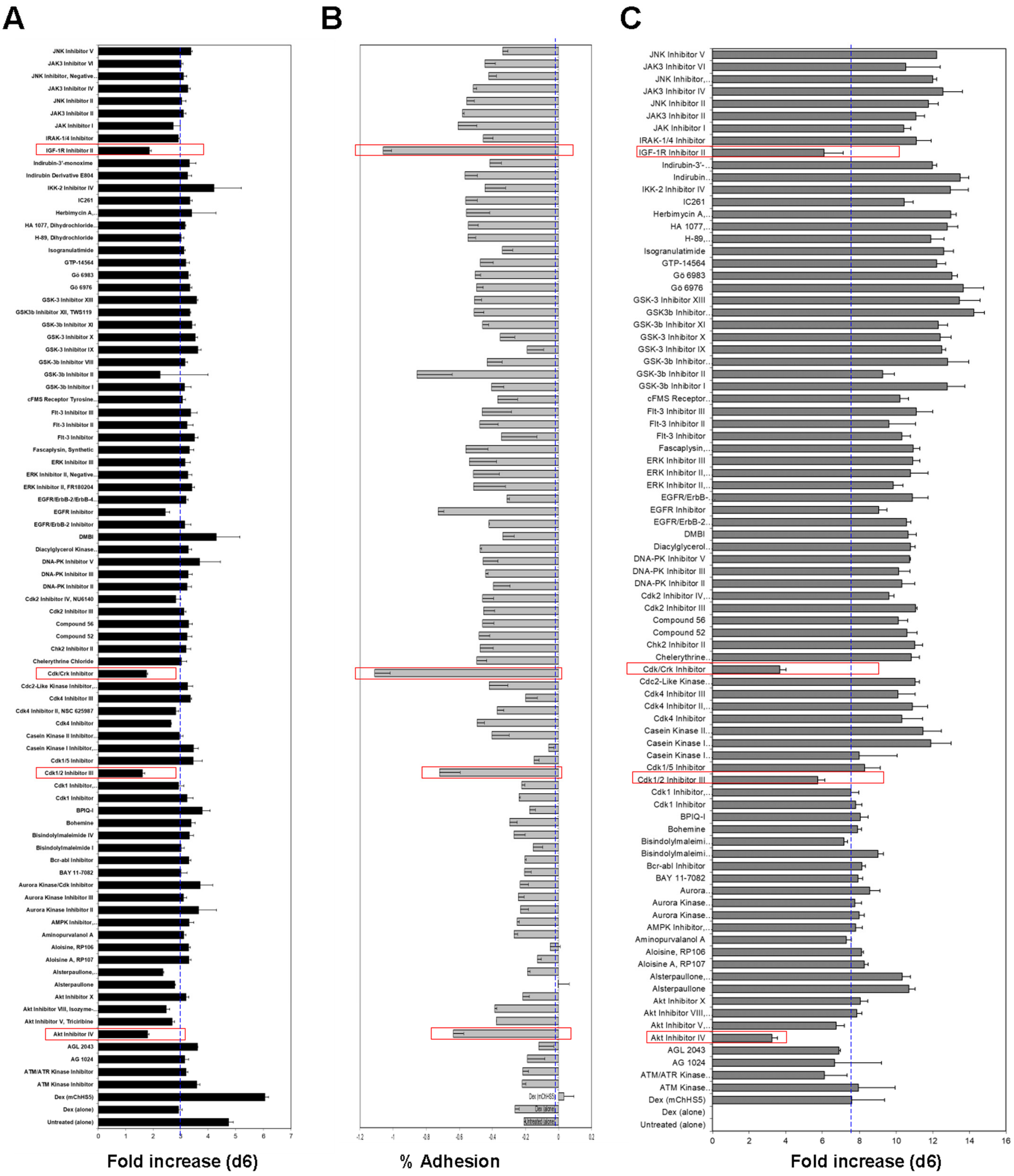
A
B
C
JNK Inhibitor V
JAK3 Inhibitor VI
JNK Inhibitor, Negative...
JAK3 Inhibitor IV
JNK Inhibitor II
JAK3 Inhibitor II
JAK Inhibitor I
IRAK-1/4 Inhibitor
IGF-1R Inhibitor II
Indirubin-3'-monoxime
Indirubin Derivative E804
IKK-2 Inhibitor IV
IC261
Herbimycin A...
HA 1077, Dihydrochloride...
H-89, Dihydrochloride
Isogranulatimide
GTP-14564
Gö 6983
Gö 6976
GSK-3 Inhibitor XIII
GSK3b Inhibitor XII, TWS119
GSK-3b Inhibitor XI
GSK-3 Inhibitor X
GSK-3 Inhibitor IX
GSK-3b Inhibitor VIII
GSK-3b Inhibitor II
GSK-3b Inhibitor I
cFMS Receptor Tyrosine...
Flt-3 Inhibitor III
Flt-3 Inhibitor II
Flt-3 Inhibitor
Fascaplysin, Synthetic
ERK Inhibitor III
ERK Inhibitor II, Negative...
ERK Inhibitor II, FR180204
EGFR/ErbB-2/ErbB-4...
EGFR Inhibitor
EGFR/ErbB-2 Inhibitor
DMBI
Diacylglycerol Kinase...
DNA-PK Inhibitor V
DNA-PK Inhibitor III
DNA-PK Inhibitor II
Cdk2 Inhibitor IV, NU6140
Cdk2 Inhibitor III
Compound 56
Compound 52
Chk2 Inhibitor II
Chelerythrine Chloride
Cdk/Crk Inhibitor
Cdc2-Like Kinase Inhibitor...
Cdk4 Inhibitor III
Cdk4 Inhibitor II, NSC 625987
Cdk4 Inhibitor
Casein Kinase II Inhibitor...
Casein Kinase I Inhibitor...
Cdk1/5 Inhibitor
Cdk1/2 Inhibitor III
Cdk1 Inhibitor...
Cdk1 Inhibitor
BPIQ-I
Bohemine
Bisindolylmaleimide IV
Bisindolylmaleimide I
Bcr-abl Inhibitor
BAY 11-7082
Aurora Kinase/Cdk Inhibitor
Aurora Kinase Inhibitor III
Aurora Kinase Inhibitor II
AMPK Inhibitor...
Aminopurvalanol A
Aloisine, RP106
Aloisine A, RP107
Alsterpaullone...
Alsterpaullone
Akt Inhibitor X
Akt Inhibitor VIII, Isozyme-...
Akt Inhibitor V, Triciribine
Akt Inhibitor IV
AGL 2043
AG 1024
ATM/ATR Kinase Inhibitor
ATM Kinase Inhibitor
Dex (mChHS5)
Dex (alone)
Untreated (alone)
0 1 2 3 4 5 6 7
Fold increase (d6)
Dex (mChHS5)
Dex (alone)
Untreated (alone)
-1.2 -1 -0.8 -0.6 -0.4 -0.2 0 0.2
% Adhesion
JNK Inhibitor V
JAK3 Inhibitor VI
JNK Inhibitor,...
JAK3 Inhibitor IV
JNK Inhibitor II
JAK3 Inhibitor II
JAK Inhibitor I
IRAK-1/4 Inhibitor
IGF-1R Inhibitor II
Indirubin-3'-...
Indirubin...
IKK-2 Inhibitor IV
IC261
Herbimycin A,...
HA 1077,...
H-89,...
Isogranulatimide
GTP-14564
Gö 6983
Gö 6976
GSK-3 Inhibitor XIII
GSK3b Inhibitor...
GSK-3b Inhibitor XI
GSK-3 Inhibitor X
GSK-3 Inhibitor IX
GSK-3b Inhibitor...
GSK-3b Inhibitor II
GSK-3b Inhibitor I
cFMS Receptor...
Flt-3 Inhibitor III
Flt-3 Inhibitor II
Flt-3 Inhibitor
Fascaplysin,...
ERK Inhibitor III
ERK Inhibitor II,...
ERK Inhibitor II,...
EGFR/ErbB-...
EGFR Inhibitor
EGFR/ErbB-2...
DMBI
Diacylglycerol...
DNA-PK Inhibitor V
DNA-PK Inhibitor III
DNA-PK Inhibitor II
Cdk2 Inhibitor IV,...
Cdk2 Inhibitor III
Compound 56
Compound 52
Chk2 Inhibitor II
Chelerythrine...
Cdk/Crk Inhibitor
Cdc2-Like Kinase...
Cdk4 Inhibitor III
Cdk4 Inhibitor II,...
Cdk4 Inhibitor
Casein Kinase II...
Casein Kinase I...
Casein Kinase I...
Cdk1/5 Inhibitor
Cdk1/2 Inhibitor III
Cdk1 Inhibitor,...
Cdk1 Inhibitor
BPIQ-I
Bohemine
Bisindolylmaleimi...
Bisindolylmaleimi...
Bcr-abl Inhibitor
BAY 11-7082
Aurora...
Aurora Kinase...
Aurora Kinase...
AMPK Inhibitor,...
Aminopurvalanol A
Aloisine, RP106
Aloisine A, RP107
Alsterpaullone,...
Alsterpaullone
Akt Inhibitor X
Akt Inhibitor VIII,...
Akt Inhibitor V,...
Akt Inhibitor IV
AGL 2043
AG 1024
ATM/ATR Kinase...
ATM Kinase...
Dex (mChHS5)
Dex (alone)
Untreated (alone)
0 2 4 6 8 10 12 14 16
Fold increase (d6)

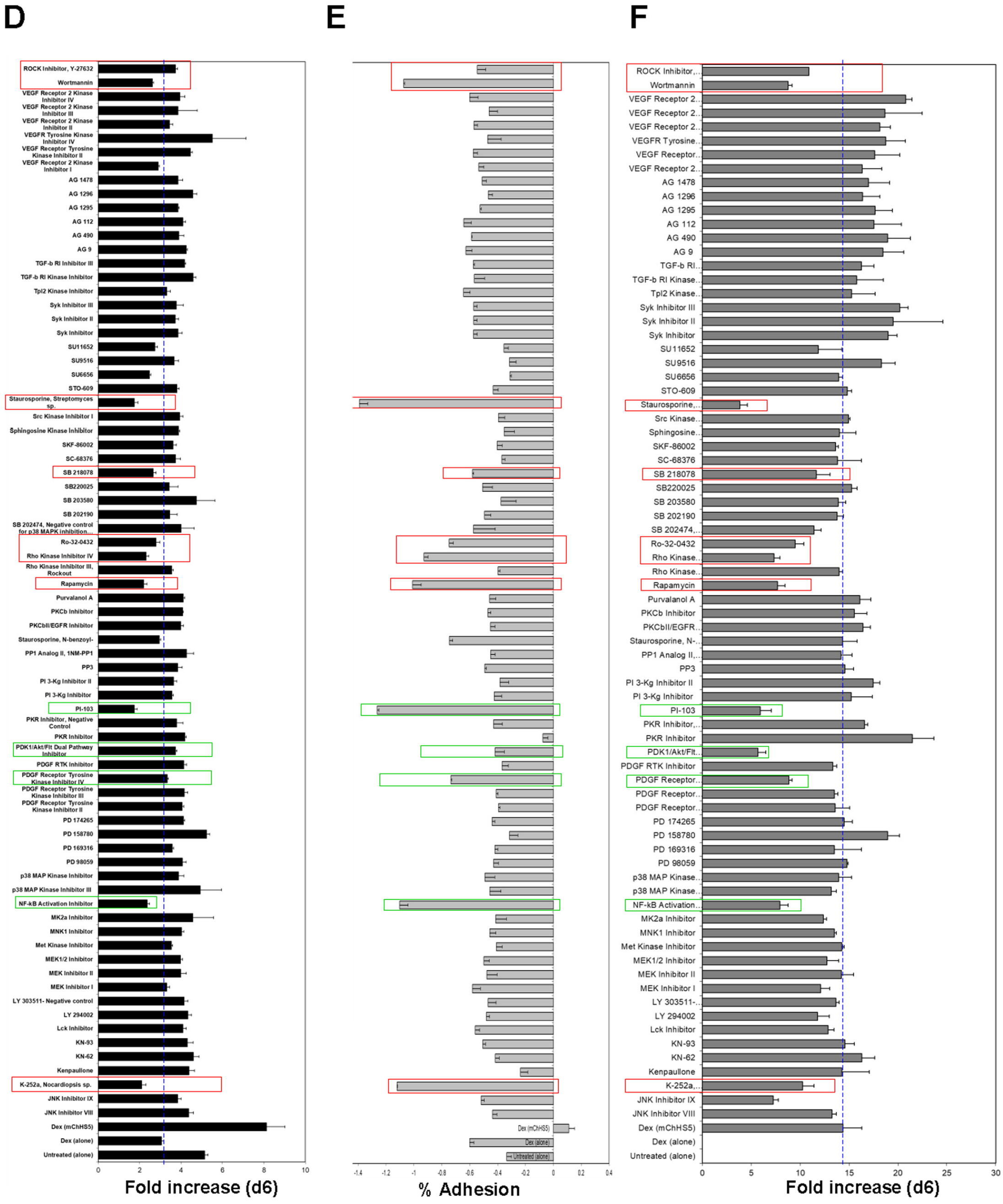

Figure S1. Analysis of the efficacy of compounds in combination with dexamethasone (dex) to overcome BM-mesenchymal cell cytoprotection of MM cells against dexamethasone. (A, D) Analysis of the proliferation (fold increase in cell numbers in 6 days) of eGFP-MM1.S cells cultured in the presence of mCherry-HS5 and treated with 500 nM dexamethasone (dex) alone or in combination with the compounds from the Merck Library I plate. As positive control for the response to dexamethasone, eGFP-MM1.S were treated with dexamethasone in monoculture (alone). The impact of dexamethasone on eGFP-MM1.S cells cultured alone was considered the positive control; (B, E) Percentage of eGFP-MM1.S cells adhered on mCherry-HS5 cells; and (C, F) Analysis of the proliferation (fold increase in cell numbers in 6 days) of mCherry-HS5 cells cultured in the presence of eGFP-MM1.S. The blue dashed lines represent the upper threshold value to determine drug efficacy. The red and green squares encricle hits that inhibit cytoprotection against dexamethasone common or divergent in mCherry-HS5 and OCs, respectively.

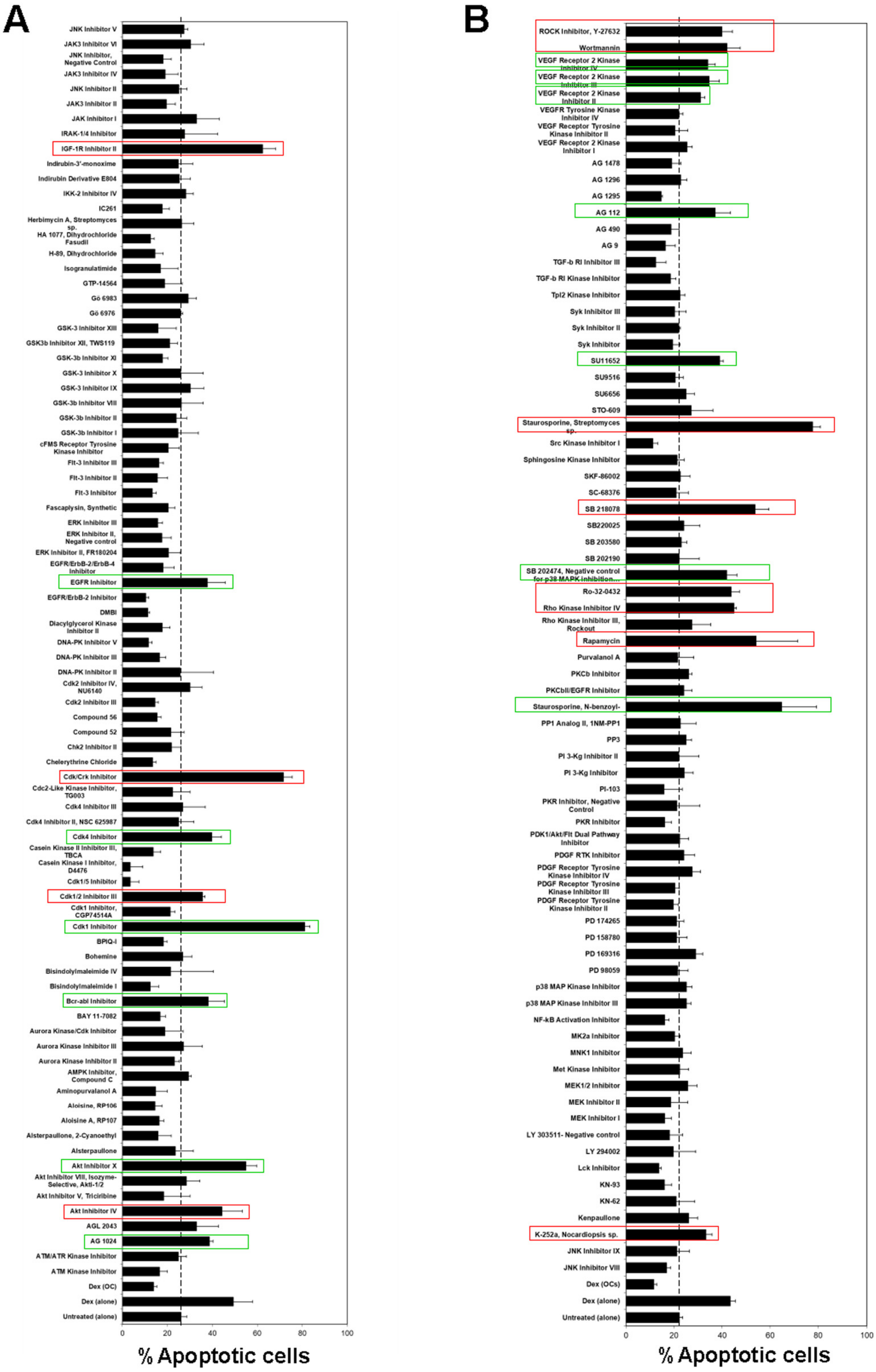

Figure S2. Analysis of the efficacy of compounds in combination with dexamethasone (dex) to overcome OC-mediated cytoprotection of MM cells against dexamethasone. (A, B) Percentage of apoptotic cells (Annexin V positive) in cultures of eGFP-MM1.S co-cultured with osteoclasts derived from MM patients and treated with dexamethasone (dex) alone or in combination with the compounds from the Merck Library I plate. The impact of dexamethasone on eGFP-MM1.S cells cultured alone was considered the positive control. The blue dashed lines represent the upper threshold value to determine drug efficacy. The red and green squares encricle hits that inhibit

cytoprotection against dexamethasone common or divergent in mCherry-HS5 and OCs, respectively.

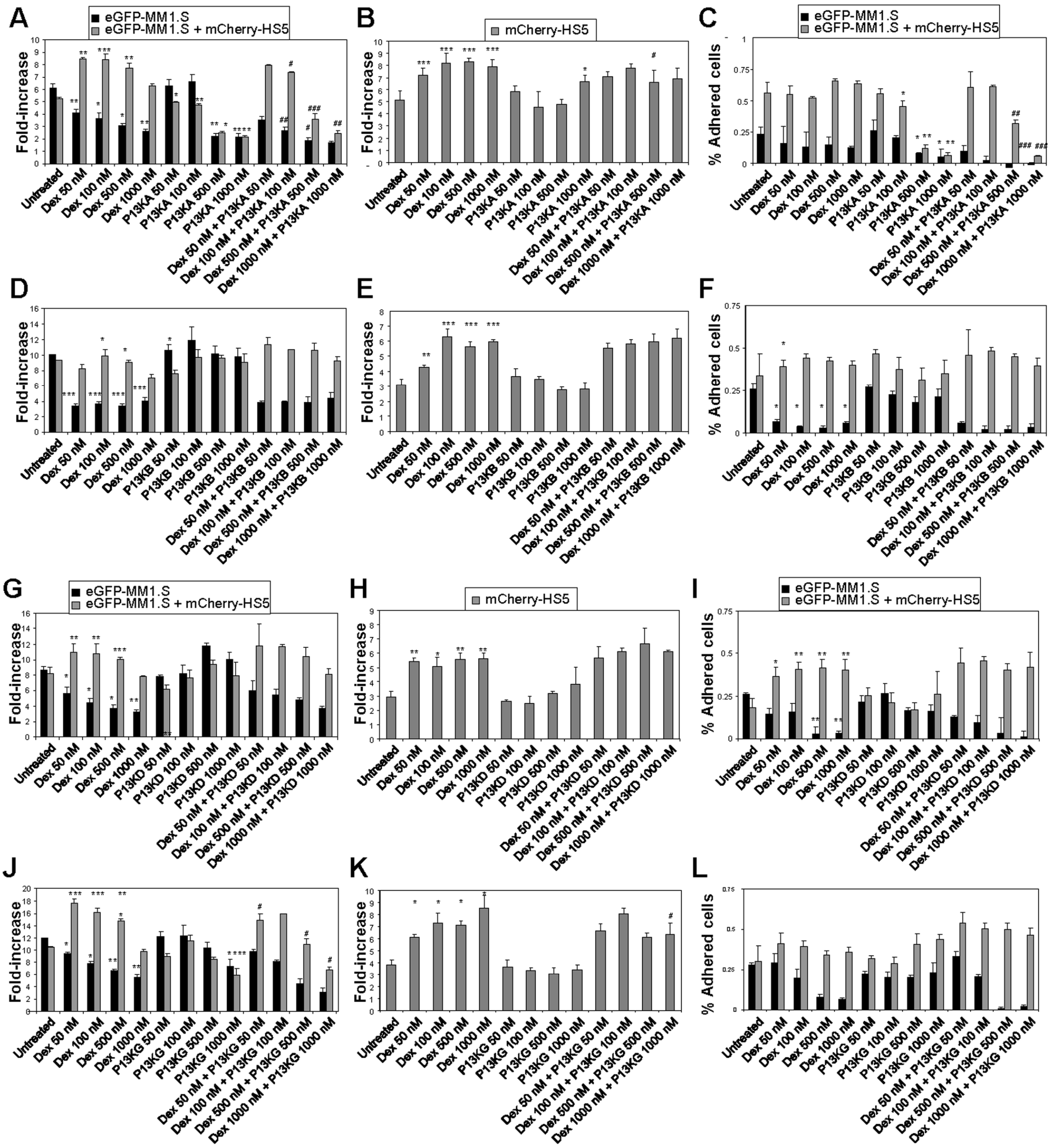

Figure S3. Analysis of the efficacy of inhibiting individual PI3K Class I isoforms to overcome BM mesenchymal cell-mediated cytoprotecion of MM cells against dexamethasone (A, D, G, J) Fluorescent-based analysis of proliferation of eGFP-MM1.S cells alone or in the presence of mCh-HS5 cells after 6 days of culture analysed by fluorimetry; (B, E, H, K) Fluorescent-based analysis of proliferation of mCherry-expressing HS5 stromal cells with MM cells; (C, F, I, L) Percentage of adhered eGFP-MM1.S cells. Cells were treated with drugs specific for the PI3K isoforms PI3K (PI3KA), (PI3KB), (PI3KD) or (PI3KG). * $p < 0.05$;** $p < 0.005$;*** $p < 0.001$ with respect to Untreated under the same experimental condition (alone or in co-culture with mCherry-HS5; # $p < 0.05$;## $p < 0.005$;### $p < 0.001$with respect to Dex treatment, two-way ANOVA test.

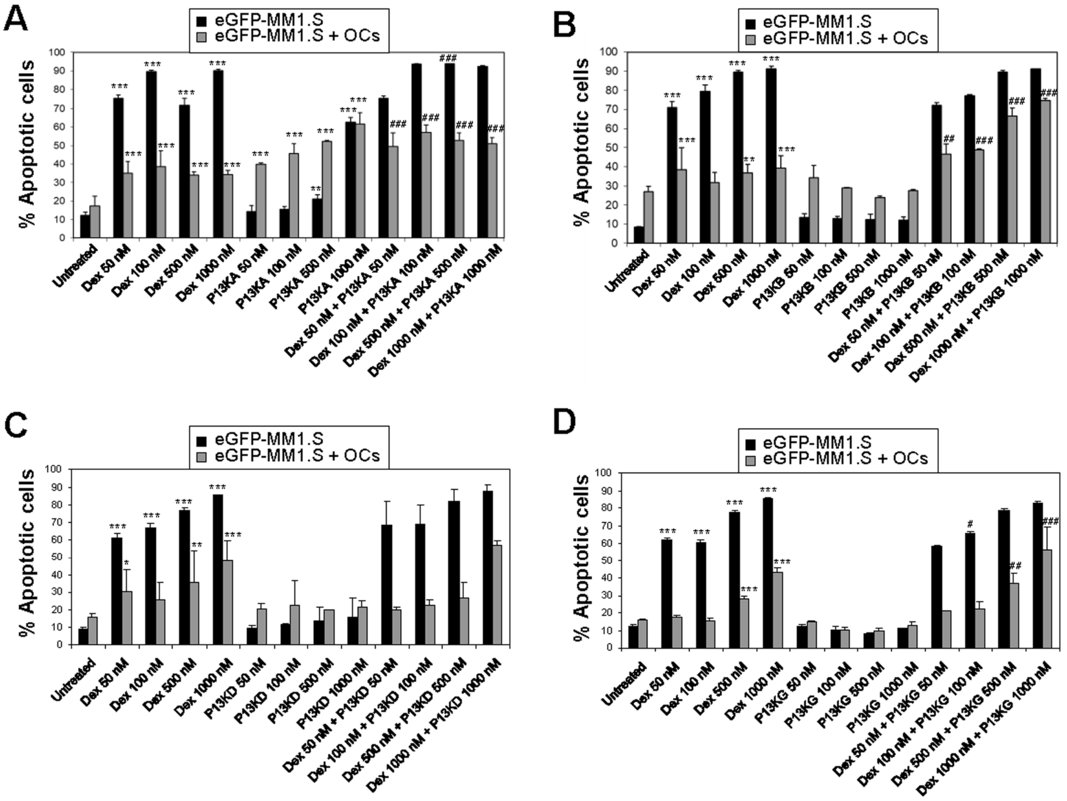

Figure S4. Analysis of the efficacy of inhibiting individual PI3K Class I isoforms to overcome OC-mediated cytoprotecion of MM cells against dexamethasone. Percentage of apoptotic cells (Annexin V positive) in cultures of eGFP-MM1.S co-cultured with osteoclasts derived from MM patients and treated with dexamethasone (dex) alone or in combination with isoforms (A) PI3K (PI3KA); (B) (PI3KB); (C) (PI3KD); and (D) (PI3KG). * p <0.05;** p < 0.005;*** p < 0.001 with respect to Untreated under the same experimental condition (alone or in co-culture with mCherry-HS5; # p < 0.05;## p < 0.005;### p < 0.001with respect to Dex treatment, two-way ANOVA test.
